# Rank, job stress, psychological distress and physical activity among military personnel

**DOI:** 10.1186/1471-2458-13-716

**Published:** 2013-08-03

**Authors:** Lilian Cristina X Martins, Claudia S Lopes

**Affiliations:** 1 Department of Epidemiology, Institute of Social Medicine, Rio de Janeiro State University (IMS/UERJ), Rio de Janeiro, Brazil; 2 Postgraduate Department/Brazilian Army School of Physical Education (EsEFEx), Escola de Educação Física do Exército-Seção de Pós-Graduação, Av. João Luíz Alves, S/Nr Urca, Rio de Janeiro, RJ CEP 22291-090, Brazil

**Keywords:** Physical activity, Mental health, Job stress, Common mental disorders, Military personnel

## Abstract

**Background:**

Physical fitness is one of the most important qualities in armed forces personnel. However, little is known about the association between the military environment and the occupational and leisure-time dimensions of the physical activity practiced there. This study assessed the association of rank, job stress and psychological distress with physical activity levels (overall and by dimensions).

**Methods:**

This a cross-sectional study among 506 military service personnel of the Brazilian Army examined the association of rank, job stress and psychological distress with physical activity through multiple linear regression using a generalized linear model.

**Results:**

The adjusted models showed that the rank of lieutenant was associated with most occupational physical activity (β = 0.324; CI 95% 0.167; 0.481); “high effort and low reward” was associated with more occupational physical activity (β = 0.224; CI 95% 0.098; 0.351) and with less physical activity in sports/physical exercise in leisure (β = −0.198; CI 95% −0.384; −0.011); and psychological distress was associated with less physical activity in sports/exercise in leisure (β = −0.184; CI 95% −0.321; −0.046).

**Conclusions:**

The results of this study show that job stress and rank were associated with higher levels of occupational physical activity. Moreover job stress and psychological distress were associated with lower levels of physical activity in sports/exercises. In the military context, given the importance of physical activity and the psychosocial environment, both of which are related to health, these findings may offer input to institutional policies directed to identifying psychological distress early and improving work relationships, and to creating an environment more favorable to increasing the practice of leisure-time physical activity.

## Background

In modern societies, although it is established knowledge that physical activity is beneficial to physical and mental health and, in some countries, policies are in place to increase it, levels of physical activity have decreased over time both in high-income countries [1], and in medium- and low-income countries [2]. In addition to factors classically associated with the practice of physical activity, such as age, gender, schooling and socio-economic status [3,4], other factors, such as changes in work processes, the incorporation of new technologies into people’s daily lives, the lack of time available for leisure, and the stress of daily living, have been shown to impact on levels of physical activity [2,5].

In the past 50 years, Brazil has undergone major demographic, economic and social changes, including a rapid process of urbanization accompanied by considerable changes in traditional employment sources and work processes. The important technological advances that have occurred in various fields have contributed to changing the nature of work, including a reduction in physical demands and increased psychological demands [6]. These changes have been considered important sources of stress and psychological problems, besides reducing the practice of physical activity.

The literature shows that physical activity, when practiced regularly, is associated with lower risk of cardiovascular disease, osteoporosis, diabetes and cancer [7,8], as well as fostering better mental health: people who engage regularly in physical activity display better quality of life and mood [9]. In addition, there is evidence that physical fitness is related to stress. Individuals with good cardio respiratory fitness are able to recover more quickly both physiologically and in the subjective dimension of the emotions [10]. Rimmele et al. [11] found that, when exposed to stressful stimuli, men with better cardio respiratory fitness displayed lower cortisol levels, better heart rate response, more calmness, better mood and a tendency to lower anxiety states than unfit men. To the authors these results suggest that physical activity may produce a protective effect against diseases related to psychological stress. At this point, it is important to highlight that stress causes a variety of health problems, and the most common and unavoidable stressors are those connected with work, lifestyle, stressful life events, and highly demanding occupations [12].

A considerable number of studies show that job stress has significant impact on individual health [13], but few studies have investigated the association between stress at work and health behaviors, particularly the practice of physical activity. Hellerstedt & Jeffery [14] formulated a theory that highly demanding work can attenuate workers’ willingness or ability to engage in regular physical activity and other types of physical activity. Corroborating that hypothesis, a study with 46,573 participants in Finland showed an inverse association between job stress and the practice of physical activity in leisure [15].

Researchers investigating the amount of physical activity practiced sought initially to understand better how health outcomes relate to total energy expenditure in individuals’ daily activities (habitual physical activity), which can be measured directly (through equipment such as pedometers or accelerometers) and indirectly (through self-reported questionnaires) [16]. More recently, aiming to improve precision, researchers have been concerned with the distinct role that occupational physical activity, acting quite differently from physical activity during leisure time, can have in the health of individuals. Prospective studies showed that while the physical activity in leisure time has a protective effect against death from cardiovascular diseases, the same does not happen with occupational physical activity [17,18]. These results underline the complexity of studying the health of the whole human being and contribute to theories of how physical activity benefits mental and physical health in various ways [19]. According to these theories physical activity fosters distraction (diverting attention from unfavorable stimuli, which leads to improved mood during and after exercise), self-efficacy (afforded by the challenging activity of exercise, because the ability to be involved on a regular basis should lead to improved mood and self-confidence), social interaction (physical activity is commonly associated with social relationships, as well as mutual support between individuals involved in the activity). These aspects play important roles in the effects of exercise on both mental health [19] and physical health, pointing to profound and complex interactions between mental and physical states. The positive affective states common during physical activity can be observed to associate with health-related neuroendocrine, cardiovascular and inflammatory processes [20].

The relationship between stress at work and mental health has been subject to considerable investigation, and the literature shows that an unfavorable work environment is associated with greater prevalence of mental disorders [6,21] and that both can affect the practice of physical activity [22,23]. There is abundant literature concerning the direction of the association, showing physical activity benefits mental health, and each day brings further evidence [24-26]. However, there has been little investigation of the influence of mental health on physical activity. Mental disorders can lead to physical inactivity due to psychological and physical symptoms. Patients with depressive symptoms show changes including social isolation, fatigue, low motivation and reduced exercise capacity [1,27]. Among the few studies identified is a recent narrative review, which concluded that the prevalence of low levels of physical activity among persons with severe mental disorders is much higher than in the general population [28]. Only one population-based study on the topic was identified. Focusing on symptoms of depression and nervousness (psychological distress) it was conducted on a sample of 5,708 participants. The results showed that men and women with high levels of psychological distress were more likely to be physically inactive. The odds ratios were 1.30 among men (95% confidence interval of 1.07 to 1.49) and 1.31 among women (95% CI 1.09 -1.55) [29].

Physical activity forms an integral part of military life, because physical training is designed to improve the troop’s physical fitness, and is always encouraged and even required. Military personnel’s rank-related occupational characteristics may be associated with the occurrence of job stress and psychological distress [30]. In this type of population, poor physical fitness is associated with low productivity and greater absenteeism [31].

The Brazilian army conducts a physical fitness test, which includes evaluating cardio respiratory fitness, upper limb and trunk strength, and other physical attributes. It is applied three times a year, and the results are taken into account for career promotion purposes. Although days, times, venues and materials for military physical training are provided at the barracks, the amount of physical activity practiced by each soldier is an individual decision. During these hours, military personnel are entitled to choose what activity they will practice, for how long per session and how many times a week.

The literature on the practice of physical activity in the military includes several studies focusing mainly on how factors ranging over aspects of physical fitness training and related nutritional concerns associate with cardio respiratory fitness and with physical health [32-36]. Despite the importance of physical activity among service personnel, little research addressed the associated factors, and no study was found to have evaluated the association of job stress and mental health with physical activity in populations of this kind. In this context, in agreement with the findings of the few studies identified on the subject, our hypothesis is that, among military personnel, both job stress and psychological distress are associated with lower levels of physical activity in leisure, despite the imperative need to engage in activities that lead to good physical fitness. On the other hand the explanatory variables are related to higher levels of occupational physical activity.

The aim of this study was to investigate how rank, job stress and psychological distress associated with physical activity among military personnel.

## Methods

### Study design and population

This cross-sectional study was conducted among all the military personnel of a Brazilian army directorate and the military organizations subordinated to it. The complement was 654 service personnel, of whom 68 (10.40%) were excluded for being away from the directorate indefinitely on missions. The eligible population was 586 service personnel.

### Measurements

The questionnaires used were self-administered, and divided into blocks by socio-economic and demographic characteristics, occupational details, and levels of physical activity, job stress and psychological distress.

#### ***Physical activity***

Level of physical activity (outcome) was evaluated using the Baecke Questionnaire [37] validated in Brazil [38,39]. The questionnaire comprises 16 questions covering three dimensions: “occupational physical activity” (OPA) – 8 questions; “sports/exercise in leisure time” (SEL) – 4 questions; and “other physical activities in leisure and locomotion” (PALL) – 4 questions. A score is calculated for each dimension, and the total score (TS), which is the sum of the three dimensions, is calculated for overall habitual physical activity.

#### ***Job stress***

Job stress was evaluated using the effort-reward imbalance (ERI) model [40]. This was translated, adapted and validated in Brazil by Chor et al. [41], and showed appropriate levels of reproducibility for the three facets (effort, reward, and over-commitment). The estimated interclass correlation coefficients were above 0.76 and the internal consistency (Cronbach’s alpha) values estimated for the facets were 0.68 for effort, 0.78 for reward, and 0.78 for over-commitment [41].

The instrument comprises 23 questions covering three facets: effort, reward and over-commitment. Effort refers to the extrinsic effort required from the employee in response to demanding aspects of the work environment; it expresses the subjective perception of workload (6 questions). Reward refers to aspects beyond financial remuneration that include job stability, career opportunities and esteem from chief/colleagues (11 questions). Over-commitment expresses whether coping strategies have been used exhaustively, and thus reflects continued and frustrated efforts, which are related to negative feelings (6 questions). The results are expressed as scores [40].

High effort in combination with low reward in the work environment represents a reciprocity deficit between “costs” and “gains” that leads to a state of active distress and evokes negative emotions. This is the most unfavorable psychosocial situation at work. According to the methodology of the model [40,42-44], quadrants were constructed for job stress, and the scores for the facets of the ERI model were dichotomized as follows: the two lower tertiles of scores were coded to 0 (low) and the upper tertile, to 1 (high). The quadrants thus established were: “high effort and low reward”, “high effort and high reward”, “low effort and high reward” and “low effort and low reward”.

#### ***Psychological distress***

Psychological distress was measured using the validated Brazilian version [45] of the General Health Questionnaire-12 items (GHQ-12) [46], which showed a sensitivity of 85% and a specificity of 71%. Scores for individual items were coded as absent or present (0 or 1) and then added. Total scores of 3 or more (out of 12) were classified as representing psychological distress (case) according to the methodology of the construct.

#### ***Rank***

Rank was categorized as follows: “Superior officers and Captains” (captain, major, lieutenant-colonel and colonel), “Lieutenants”, “Sergeant-Majors and Sergeants” and “Corporals and Privates”.

#### ***Covariates***

The covariates were age, education, income, marital status, and lifestyle variables. Income was calculated as per capita family income, i.e., total family income divided by the number of family members living on that income, and was then categorized in Brazilian minimum wages. Lifestyle variables were alcohol consumption and smoking habits. The covariates were considered as adjustment factors for associations among the variables of interest.

#### ***Instrument reliability and data collection***

In order to guarantee the quality of the information in the questionnaires, pre-test, pilot, reliability and reproducibility tests were conducted on the instruments used. Levels of agreement were in excess of 70% in each of the instruments. The authors performed data collection between October 2009 and March 2010 at the directorate and at its subordinate military organizations.

### Statistical analysis

Exploratory and descriptive analyses were performed. Reference categories for the subsequent analysis were established as follows: for job stress the reference category, which represents a more favorable psychosocial work environment, is “low effort and high reward”; and, for psychological distress, the reference category was the absence of symptoms (“No”). In order to evaluate how job stress, psychological distress and rank associate with the physical activity scores (OPA, SEL, PALL and TS), bivariate analysis was performed by simple linear regression via generalized linear models.

To assess the associations among the variables of interest (outcome, explanatory variables and covariates), the multiple linear regression method was used, with one model being designed for each of the scores (Model 1). The associations were adjusted for the socio-economic and demographic characteristics: age, schooling, income and marital status. For that purpose, multiple linear regression via generalized linear models (Model 2) was performed, and 95% confidence intervals (CI) were calculated to assess the accuracy of the findings. Records with missing data were excluded from the analyses.

The data were input into MSAccess 2000, and the statistical analyses were performed using the R platform [47].

### Ethical principles

All participants signed a declaration of free and informed consent. The Ethics Committee of the Institute of Social Medicine, at Rio de Janeiro State University, approved the study.

## Results

Of the 586 eligible candidates, 46 (7.85%) refused to take part (their distribution showed no pattern among them). Women were withdrawn from the study because of their small number (34). The total number of participants was thus 506. Their mean age was 29 (±9.77) years. Most were single (49.8%), had per capita income of up to five minimum wages (64.4%), higher schooling (62.3%), and were childless (66.2%).

Few participants failed to complete the outcome questionnaire properly, resulting in missing data by dimension as follows: OPA, 5 (0.99%); SEL, 6 (1.19%); PALL, 7 (1.38%); and TS, 8 (1.58%).

Table 1 shows the summary measures of physical activity (total score and dimensions). The data display normal or approximately normal distribution with little variability.

**Table 1:** Descriptive statistics on physical activity among the military (N = 506)

**Dimension of physical activity**	**N**	**Mean**	**Median**	**SD**	**Min.**	**Max.**
Total score	498	9.70	9.75	1.21	5.75	13.00
Occupational physical activity (OPA)	501	3.15	3.13	0.53	1.75	4.75
Sports/physical exercise (SEL)	500	3.67	3.75	0.70	1.50	5.00
Other physical activities in leisure and locomotion (PALL)	499	2.88	3.00	0.64	1.00	4.50

Fifty-two percent of the directorate personnel viewed their work environment as favorable (“low effort and high reward”), and 16.2% showed signs of job stress (“high effort and low reward”).

Table 2 shows the results of the bivariate analysis to evaluate how job stress, psychological distress and rank associate with each of the physical activity scores. Only rank associated with higher total scores (TS) (lieutenants: β = 0.531 CI 95% 0.211; 0.851; and corporals and privates: β = 0.680 CI 95% 0.389; 0.970). Job stress (β = 0.405 CI 95% 0.279; 0.531), psychological distress (β = 0.152 CI 95% 0.055; 0.250) and rank (lieutenants: β = 0.553 CI 95% 0.422; 0.684; and corporals and privates: β = 0.412 CI 95% 0.294; 0.531) associated with higher occupational physical activity (OPA) scores. Only rank (corporals and privates: β = 0.328 CI 95% 0.173; 0.483) displayed an association with “other physical activities in leisure and locomotion” (PALL).

**Table 2:** **Bivariate analysis for associations of job stress**, **psychological distress and rank with physical activity**

**Dimensions of physical activity and characteristics**	**β**	**(CI 95%)**
**TS**^**a**^		
*Job stress by quadrant*		
Low effort/high reward^b^	-	
Low effort/low reward	0.191	(−0.104; 0.486)
High effort/high reward	0.186	(−0.133; 0.504)
High effort/low reward	0.152	(−0.151; 0.455)
*Psychological distress*		
No^b^	-	
Yes	−0.190	(−0.336; 0.118)
*Rank*		
Field officers and captains^b^	-	
Lieutenants	**0.531**	**(0.211; 0.851)**
Sergeant-majors and sergeants	−0.089	(−0.411; 0.239
Corporals and privates	**0.680**	**(0.389; 0.970)**
**OPA**^**c**^		
*Job stress by quadrant*		
Low effort/high reward^b^	-	
Low effort/low reward	0.072	(−0.050; 0.194)
High effort/high reward	**0.235**	**(0,102; 0.367)**
High effort/low reward	**0.405**	**(0.279; 0531)**
*Psychological distress*		
No^b^	-	
Yes	**0.152**	**(0.055; 0.250)**
*Rank*		
Field officers and captains^b^	-	
Lieutenants	**0.553**	**(0.422; 0.684)**
Sergeant-majors and sergeants	0.057	(−0.075; 0.190)
Corporals and privates	**0.412**	**(0.294; 0.531)**
**SEL**^**d**^		
*Job stress by quadrant*		
Low effort/high reward^b^	-	
Low effort/low reward	−0.014	(−0.182; 0.154)
High effort/high reward	−0.022	(−0.205; 0.160)
High effort/low reward	**−0.209**	**(−0.383; −0.036)**
*Psychological distress*		
No^b^	-	
Yes	**−0.171**	**(−0.301; −0.042)**
*Rank*		
Field officers and captains^b^	-	
Lieutenants	0.030	(−0.159; 0.219)
Sergeant-majors and sergeants	**−0.256**	**(−0.448; −0.065)**
Corporals and privates	−0.057	(−0.228; 0.115)
**PALL**^**e**^		
*Job stress by quadrant*		
Low effort/high reward^b^	-	
Low effort/low reward	0.110	(−0.046; 0.266)
High effort/high reward	−0.032	(−0.201; 0.137)
High effort/low reward	0.049	(−0.210; 0.112)
*Psychological distress*		
No^b^		
Yes	−0.089	(−0.209; 0.031)
*Rank*		
Field officers and captains^b^	-	
Lieutenants	−0.052	(−0.224; 0.119)
Sergeant-majors and sergeants	0.102	(−0.072; 0.276)
Corporals and privates	**0.328**	**(0.173; 0.483)**

Association of job stress, psychological distress and rank with physical activity – results of the bivariate analysis, by simple linear regression via generalized linear models (2010).

β = Coefficient.

^a^TS (total score) = OPA + SEL + PALL.

^b^Reference category.

^c^OPA = occupational physical activity.

^d^SEL = physical activity in sports/physical exercise during leisure.

^e^PALL = other physical activities during leisure and in walking.

Obs.: Age was significantly associated (p < 0.05) with TS, OPA and PALL, all three decreasing with increasing age.

Table 3, showing the results of the multivariate analysis, gives the results from Model 1, which analyzed simultaneously the association among the independent variables and the dependent variables, for which models were designed for each of the physical activity scores. Note that in this data set, the ranks of “lieutenant” and “corporals and privates” continued to display a statistically significant association with higher TS, with no major alterations in the gradients. Job stress and rank continued to associate with higher levels of OPA, while psychological distress ceased to be significant. The ranks of “corporals and privates” maintained their association with PALL.

**Table 3:** **Adjusted models job stress**, **psychological distress and rank with physical activity among military personnel**

**Dimensions of physical activity and characteristics**	**Model 1**		**Model 2**	
	**β**	**(CI 95%)**	**β**	**(CI 95%)**
**TS**^**a**^				
*Job stress by quadrant*				
Low effort/high reward^b^	-		-	
Low effort/low reward	−0.005	(−0.030; 0.291)	−0.061	(−0.364; 0.241)
High effort/high reward	0.190	(−0.124; 0.505)	0.121	(−0.199; 0.442)
High effort/low reward	−0.025	(−0.334; 0.285)	−0.066	(−0.387; 0.255)
*Psychological distress*				
No^b^	-		-	
Yes	−0.223	(−0.454; 0.008)	−0.216	(−0.453; 0.020)
*Rank*				
Field officers and captains^b^	-			
Lieutenants	**0.510**	**(0.271; 0.929)**	0.281	(−0.117; 0.678
Sergeant-majors and sergeants	−0.056	(−0.384; 0.270)	−0.187	(−0.561; 0.187)
Corporals and privates	**0.723**	**(0.425; 1.034)**	0.124	(−0.486; 0.733)
**OPA**^**c**^				
*Job stress by quadrant*				
Low effort/high reward^b^	-		-	
Low effort/low reward	−0.028	(−0.145; 0.090)	−0.056	(−0.175; 0.063)
High effort/high reward	**0.195**	**(0.070; 0.321)**	**0.183**	**(0.057; 0.310)**
High effort/low reward	**0.265**	**(0.141; 0.388)**	**0.224**	**(0.098; 0.351)**
*Psychological distress*				
No^b^	-		-	
Yes	0.013	(−0.079; 0.105)	0.040	(−0.053; 0.134)
*Rank*				
Field officers and captains^b^	-			
Lieutenants	**0.511**	**(0.380; 0.643)**	**0.324**	**(0.167; 0.481)**
Sergeant-majors and sergeants	0.079	(−0.051; 0.210)	0.013	(−0.135; 0.160)
Corporals and privates	**0.310**	**(0.279; 0.521)**	0.162	(−0.078; 0.403)
**SEL**^**d**^				
*Job stress by quadrant*				
Low effort/high reward^b^	-		-	
Low effort/low reward	−0.001	(−0.180; 0.165)	0.009	(−0.167; 0.185)
High effort/high reward	−0.017	(−0.200; 0.167)	−0.030	(−0.217; 0.156)
High effort/low reward	**−0.213**	**(−0.394; −0.032)**	**−0.198**	**(−0.384; −0.011)**
*Psychological distress*				
No^b^	-			
Yes	**−0.171**	**(−0.306; −0.036)**	**−0.184**	**(−0.321; −0.046)**
*Rank*				
Field officers and captains^b^				
Lieutenants	0.112	(0.080; 0.304)	0.056	(−0.174; 0.286)
Sergeant-majors and sergeants	**−0.250**	**(−0.441; −0.060)**	−0.202	(−0.419; 0.016)
Corporals and privates	−0.007	(−0.184; 0.171)	−0.017	(−0.369; 0.334)
**PALL**^**e**^				
*Job stress by quadrant*				
Low effort/high reward^b^				
Low effort/low reward	0.010	(−0.149; 0.169)	−0.026	(−0.190; 0.137)
High effort/high reward	0.007	(−0.161; 0.176)	−0.038	(−0.211; 0.135)
High effort/low reward	−0.084	(−0.250; 0.082)	−0.103	(−0.277; 0.070)
*Psychological distress*				
No^b^			-	
Yes	−0.063	(−0.187; 0.061)	−0.078	(−0.206; 0.049)
*Rank*				
Field officers and captains^b^	-		1.	2.
Lieutenants	−0.022	(−0.198; 0.154)	−0.076	(−0.290; 0.137)
Sergeant-majors and sergeants	0.104	(−0.071; 0.280)	0.009	(−0.193; 0.211)
Corporals and privates	**0.345**	**(0.183; 0.508)**	0.025	(−0.301; 0.352)

Models adjusted to evaluated associations of job stress, psychological distress and rank with physical activity among military personnel – results of the multivariate analysis by multiple linear regression via generalized linear models.

Model 1: Simultaneous adjustment of the independent variables (job stress, psychological distress and rank).

Model 2: Simultaneous adjustment of the independent variables with socio-demographic variables (age, schooling, income and marital status).

β = Coefficient.

^a^TS (total score) = OPA + SEL + PALL.

^b^Reference category.

^c^OPA = occupational physical activity.

^d^SEL = physical activity in sports/physical exercise during leisure.

^e^PALL = other physical activities during leisure and in walking.

In Model 2 (Table 3), which evaluated simultaneously the association of physical activity with the independent variables and socio-economic and demographic variables (age, schooling, income and marital status), rank can be seen to have lost its association with higher TS levels.

After adjustments, job stress continued to be associated with higher levels of OPA (β = 0.224 CI 95% 0.098; 0.351), with no major gradient changes. The rank “corporals and privates” ceased to show an association with more OPA, while the rank of “lieutenant” continued so associated (β = 0.324 CI 95% 0.167; 0.481), even in the presence of the socio-economic and demographic variables. Psychological distress lost its initial association with more OPA.

Job stress and psychological distress maintained their associations with lower levels of SEL (β = −0.198 CI 95% -0.384; -0.011) and (β = −0.184 CI 95% -0.321; -0.046), respectively.

The rank “corporals and privates” lost its association with higher levels of PALL.

## Discussion

This study examined the relationship between physical activity and rank, job stress (as framed by the effort-reward imbalance/ERI model) and psychological distress. The main findings were that job stress was associated with higher levels of “occupational physical activity” and lower levels of “physical activity in sports / physical exercise in leisure time”. In addition, psychological distress also displayed an association with lower levels of “physical activity in sports / physical exercise during leisure”.

Siegrist [40] argues that the combination “high effort and low reward” defines the situation of chronic job stress, constituting an adverse psychosocial environment. In the present study, job stress and the rank of lieutenant showed independent associations with higher levels of “occupational physical activity” after adjustment for age, schooling, income and marital status. The “effort” dimension of the ERI model contemplates psychological aspects such as: interruptions in work, time pressure to meet demands, and others; and also contains a question eliciting perception of major physical effort. The “occupational physical activity” dimension of the Baecke Questionnaire evaluates energy expenditure in the main occupation type, and includes questions about how often respondents remain standing, walk, and carry weight, among others. It is thus possible that this dimension associates strongly with the “physical effort” item of the ERI model. Nonetheless, it has to be borne in mind that there are other psychological components that may be associated with component factors of this physical activity. For example, the items “In recent years, my job has come to demand more and more of me” and “I have a lot of responsibility in my job” (in terms of more planning and execution) may reflect more time being spent standing and walking at work. In this regard, the rank of lieutenant, which is a “sandwich category” (the lowest rank among commissioned officers and the first rank above the non-commissioned officers) and has peculiar occupational characteristics, as discussed by Martins & Lopes [42]. They explain that those holding this rank have to report to a larger number of superiors and are also often the ones directly responsible for performing the tasks, which are planned by the higher ranks but must be fulfilled to the letter by non-commissioned officers, corporals and privates. Thus, this occupational profile may lead to higher frequencies of standing and walking at work.

Psychological distress, which initially had shown an association with “occupational physical activity”, lost that association in the multivariate model. These results suggest that mental health does not influence the amount of physical activity performed in military work routines. Kim et al. [48] report that many studies have addressed the relationship between “physical activity during leisure” and “psychological distress”, but the influence of “psychological distress” on “occupational physical activity” has not yet been investigated. However, it is important to note that high levels of “occupational physical activity” are associated with higher scores for “psychological distress” in men [48].

The results for SEL in Model 2 show that both “job stress” and “psychological distress” were independently associated with less “physical activity in sports/physical exercise in leisure time” after adjustment for age, schooling, income and marital status. The coefficients in Models 1 and 2 show no differences, and the statistical association continues of the same order of magnitude.

The negative association found between “job stress” and “physical activity in sports/physical exercise in leisure time”, and the absence of an association with “other physical activities in leisure and locomotion”, independent of the covariates, are results that agree with the literature. A cohort study with 10-year follow-up conducted in a population sample (N = 7066) in Denmark found that individuals with high levels of stress were twice as likely to become physically inactive when compared with individuals with low levels of stress [49]. Mäkinen et al. [50] also found that a current history of job stress influences physical inactivity during leisure. Wijndaelea et al. [23], in a population sample study in Belgium, with 2616 participants, found that people with job stress participated less in physical activity in sports than in other activities (such as housework, gardening or in locomotion), in keeping with the findings of this study. In addition, the results found by Kouvonen et al. [15] showed that, among Finnish civil servants (n = 46573), job stress led to lower levels of physical activity in leisure time. Pointing in the same direction, Ali & Lindstrom [51], in a population sample study (n = 5180) in Sweden, also found that high demands (tension) at work are associated with lower levels of physical activity during leisure.

In this respect, Rod et al. [49] explain that stress reduces the time and energy necessary to engage in physical activity and, on the other hand, people who do engage in regular physical activity see their lives as less stressful than those who do not. As levels of physical activity were largely high in the study population, this may explain the high prevalence of military service personnel who perceived their work environment as favorable (52% not stressed). One factor that may be related to the feeling of immaterial reward is the fact that the official daily work schedule sets aside time for physical activity.

This study investigated physical activities practiced during leisure, and the instrument used for measurement took in more detailed information about leisure-time activity types, dividing them into “sports and/or exercises in leisure time” and “other physical activities in leisure and locomotion”. Our results indicate that “job stress” was associated only with less sports activities or physical exercise, and not with other leisure time physical activities, such as walking or cycling. As “physical exercise” is a subcategory of “physical activity” that is planned, structured, repetitive, and engaged in for the purpose of improving or maintaining one or more components of physical fitness [52], there is a psychological engagement necessary to maintain this kind of practice, which is in itself a challenge. On the other hand, the presence of stress showed no association with the practice of informal physical activities during leisure (cycling or walking, for example). These findings are in agreement with those of the study by Widjndaelea et al. [23]. Furthermore, longitudinal studies have shown that the effect of unfavorable work environments is to lead to a reduction or cessation of physical activity [49,53].

The presence of “psychological distress” was independently associated with less SEL after adjustment for the socio-economic and demographic, health and lifestyle variables, which agrees with the literature. The review study by Jones & O’Beney [54] noted that people with mental disorders are generally less active and more sedentary. Another study, conducted by Muhsen et al. [29] in a population-based sample of 5,708 participants in Israel, found that men and women with high levels of psychological distress displayed greater likelihood of physical inactivity. The likelihood ratios were 1.30 among the men (95% confidence interval of 1.07 to 1.49) and 1.31 among the women (CI95% 1.09-1.55).

Our results show that “job stress”, “psychological distress” and “military rank” are not associated with total score for physical activity, or with “other physical activities in leisure and locomotion”.

The exposure variables in this study (psychosocial factors) can be observed to have different effects on each of the different dimensions of physical activity. Job stress was associated, on the one hand, with higher levels of occupational physical activity (OPA) while, on the other hand, also associating with lower levels of physical activity in sports/exercise. Another important point is that psychological distress was also associated with lower levels of physical activity in sports/exercise (SEL). These findings indicate that the presence of job stress and psychological distress concomitantly would probably lead to even lower levels of such activity. The additional finding that psychosocial factors were not significantly associated with other physical activities in leisure and locomotion (PALL) may explain the lack of association with overall physical activity, which is the sum of OPA, SEL and PALL. There is limited literature available on the relationship between psychological distress and overall physical activity [48]. The same occurs in the investigation of job stress and dimensions of physical activity (overall, occupational and other physical activities in leisure and locomotion). It may be appropriate to point out that occupational physical activity is associated with higher risk of cardiovascular disease and mortality in men [18]. Moreover, it does not protect men or women from these events [17]. Nevertheless, the role of job stress in this relationship remains unclear.

Our findings indicate the need for more studies of the effects of psychosocial factors on the different dimensions of physical activity.

### Strong points of the study

The literature shows that few studies have investigated how job stress associates with levels of total, occupational and leisure-time physical activity and with mental health [49]. This study aimed to contribute to knowledge on this subject.

The first strong point of the study was the participation rate (92%), which is considered very high, and the choice of study population, which was socio-economically and demographically homogeneous, which reduced the likelihood of residual confounders. In addition, as the population sample has the same socio-economic and demographic characteristics as the Brazilian army ground forces overall [55], the results can be extrapolated to the whole Brazilian army.

Another point in favor of this study is the 1.58% information loss rate, which can be considered small and is reasonable for epidemiological studies.

Another strong point is the choice of instrument used for estimating levels of physical activity, because it covers the prior 12 months (habitual practice), and offers important information as regards the types of physical activity, typified as “occupational physical activity”, “physical activity in sports and /or physical exercise in leisure time”, and “other physical activities in leisure and locomotion”, which permitted more detailed analysis of the dimensions that make up the set of physical activities practiced habitually, and thus more minute examination of the different aspects of physical activity and their relationships with other factors that affect health.

### Limitations of the study

The proportion of women among the military personnel participating in the study (census) was initially far higher (6%) than in the army overall (1.3%), which could have distorted the results and weakened their power. Accordingly, women were excluded from the analyses. Nevertheless, our findings are still comparable to the studies cited here because most of them analyzed the outcomes stratifying by gender. This methodology has been published elsewhere [42].

One of the limitations of the study stems from its cross-sectional design, which means that temporal relationships in some associations cannot be evaluated, leaving the possibility of reverse causality. Nonetheless, our study is one of the few offering findings on the relationship of psychosocial factors with different dimensions of physical activity.

The association found between “job stress” and more “occupational physical activity” suggests that the first may be related to greater effort at work, but it was not possible in this study to determine the direction of the association. The literature shows that physical activity has a beneficial impact on perception of the work environment [56,57], and a good work environment can favorably influence the practice of physical activity [15,53]. However, it has to be considered that psychosocial factors may affect health-related behaviors, such as the practice of physical activity. As regards the association between stress in the work environment and “physical activity in sports/physical exercise in leisure time”, our findings are in agreement with those of other cross-sectional studies in the literature. Also, longitudinal studies have shown that job stress does tend to reduce or halt the practice of physical activity [49,53].

## Conclusions

In conclusion, this study showed that “military rank”, “job stress” and “psychological distress” are associated with physical activity. However, this association shows different patterns related to dimensions of physical activity. For those occupying the rank of lieutenant and classified as displaying job stress there is a positive association with “occupational physical activity”, which indicates the relationship with specific occupational characteristics. Whereas “job stress” and “psychological distress” were associated with lower levels of “sports and exercise in leisure time”, rank showed no such association. “Job stress”, “rank” and “psychological distress” were not associated with “other physical activities in leisure and locomotion”. These findings are new, as the authors did not find any other study that has addressed this issue in military personnel.

In line with the literature, this study showed that “job stress” and “psychological distress” are negatively associated with “sports and exercise in leisure time”. Since physical activity in sports / physical exercise is associated with physical fitness, and in the professional military context this is highly important, our results can help inform the development of institutional policies directed to early identification of psychological distress and contribute to improving work relationships, by creating an environment more favorable to increasing the practice of physical activity in sports and exercise.

## Competing interests

The authors declare that they have no competing interests.

## Pre-publication history

The pre-publication history for this paper can be accessed here:

http://www.biomedcentral.com/1471-2458/13/716/prepub
